# Response Characteristics of Soil Fractal Features to Different Land Uses in Typical Purple Soil Watershed

**DOI:** 10.1371/journal.pone.0122842

**Published:** 2015-04-09

**Authors:** Bang-lin Luo, Xiao-yan Chen, Lin-qiao Ding, Yu-han Huang, Ji Zhou, Tian-tian Yang

**Affiliations:** 1 College of Resources and Environment/Key Laboratory of Eco-environment in Three Gorges Region (Ministry of Education), Southwest University, Chongqing, China; 2 State Key Lab of Urban and Regional Ecology, Research Centre for Eco-Environmental Sciences, Chinese Academy of Sciences, Beijing, China; 3 Department of Civil and Environmental Engineering, University of California Irvine, Irvine, California, United States of America; Institute for Sustainable Plant Protection, ITALY

## Abstract

As a fundamental characteristic of soil physical properties, the soil Particle Size Distribution (PSD) is important in the research on soil moisture migration, solution transformation, and soil erosion. In this research, the PSD characteristics with distinct methods in different land uses are analyzed. The results show that the upper bound of the volume domain of the clay domain ranges from 5.743μm to 5.749μm for all land-use types. For the silt domain of purple soil, the value ranges among 286.852~286.966 μm. For all purple soil land-use types, the order of the volume domain fractal dimensions is *D_clay_<D_silt_<D_sand_*. However, the values of *D_silt_* and *D_sand_* in the *Pinus massoniana Lamb*, *Robinia pseudoacacia L* and *Ipomoea batatas* are all higher than the corresponding values in the *Citrus reticulate Blanco* and *Setaria viridis*. Moreover, in all the land-use types, all of the parameters in volume domain fractal dimension (D_vi_) are higher than the corresponding parameter values from the United States Department of Agriculture (D_vi_(U)). The correlation study between the volume domain fractal dimension and the soil properties shows that the intensity of correlation to the soil texture and soil organic matter has the order as: *D_silt_>D_silt_(U)>D_sand_ (U)>D_sand_* and *D_silt_>D_silt_(U)>D_sand_>D_sand_(U)*, respectively. As it is compared with all D_vi_, the *D_silt_* has the most significant correlativity to the soil texture and organic matter in different land uses of the typical purple soil watersheds. Therefore, *D_silt_* will be a potential indictor for evaluating the proportion of fine particles in the PSD, as well as a key measurement in soil quality and productivity studies.

## Introduction

The fractal theory was proposed and established by Mandelbrot (1977, 1982) [1–2], which is a method of describing systems with non-characteristic scales and self-similarity. This theory has been utilized to quantitatively describe the characteristics of the soil particle size distribution (PSD), which is important in hydrological conductivity, solution transportation, and soil erosion. Therefore, the fractal theory attracts the interests of pedologists worldwide [3–7]. In the area of the micro-field of soil science, Scott and Stephen estimated soil water retention based on fractal mathematics and further noted the limitations of the fractal method when applied to scaling the soil PSD [8–9]. The method of measuring the soil PSD was developed with the introduction of lighting-scattering technology, which was utilized to further quantitatively calculate the soil particle size distribution [10]. Almost at the same time, with the enhancement of the PSD measurement technology, the fractal theory was also developed into multifractal theory. Perfect and Kay (1993) [11] analyzed the characteristics of soil aggregate fragmentation and provided a theoretical framework in terms of the combination of fractals and multifractals to link size distribution and strength. Bittelli et al. (1999) [12] characterized the PSD using a fragmentation model based on the mass fractal dimension (D_m_) in three domains: clay, silt, and sand. Later, Prosperinin (2008) [13] systematically described the characteristics of PSD in the Umbria region of Italy using the fractal mathematics method. Unlike traditional factors used to determine the characteristics of hydraulic properties of soil, Eran (2009) and Lalit (2010) applied the fractal theory to predict the soil hydraulic properties through the measurement of PSD. Some scholars also applied the multifractal model on PSD. Posadas et al. (2001, 2003, 2010) [14–16] characterized the soil particle distribution by applying the multifractal method, interpreted the relationship between the soil textural features and parameters of the multifractal model, and described the soil pore system based on the multifractal method. The studies from Posadas et al. (2001, 2003, 2010) [14–16] have improved the integration of spatial properties of the soil pore system. In addition, Miguel (2002) applied the multifractal model to characterize the soil volume-size distribution. Caniego (2005) [17] also focused on the soil spatial properties in terms of organic matter and electrical conductivity using multifractal theory.

The fractal and multifractal models became even more important for quantitatively describing the characteristics and behaviors of the soil particles with the development of laser diffraction technology and the stochastic methods based on probability theories, which is more reasonable in describing the features of the soil solid phase [18]. These developments provide a necessary environment for the movement of soil liquid and gas phases, all of which strongly affect the eco-hydrological processes. Soil fractal dimension is an important parameter to study soil structure, soil texture and soil erosion. It has an important theoretical value and practical significance to study the soil fractal dimension in the purple soil distributed region.

In China, Yang et al. (1993) [19] introduced a calculation theory concerning the soil PSD mass fractal dimension (D_m_). Huang (2002, 2005) [20–21] analyzed the relationship between the soil particle size mass distribution and the soil compartments, including the clay, silt, and sand contents. The results from Huang (2002, 2005) [20–21] indicated that the mass-based fractal dimension of the soil PSD could be used to predict the soil water retention properties. Huang further fitted the relationship between the fractal dimension and soil texture and evaluated the fractal distribution with a water retention curve. However, Martin and Montero (2002) [22] questioned the assumption that soil particles with different sizes have the same density in the D_m_ calculations. In order to avoid this issue, another approach to calculate the soil particle fractal dimension and the volume fractal dimension D_v_, was proposed by Wang et al. (2005) [23]. Since then, fractal theory has become well-developed, as pointed out by Yang (2008) [24], in which the comparison and analysis of the mass fractal dimension D_m_ and the volume fractal dimension D_v_ were presented. The fractal and multifractal theory have been also widely applied to various topics in soil science, including land use, desertification, and the characteristics of specific soil particles [7, 24–27]. Wang (2008) [28] applied the multifractal theory to analyze the effect of some parameters in the multifractal model on the different land-use types in the Loess Plateau of China, and Liu (2009) [29] focused on the effect of the fractal features of soil PSD on different plant communities in Chinese forests. With regards to the studies mentioned above, very few studies have been conducted on the response of the PSD fractal features to the different land use types in the small purple soil watershed in southwestern China. Zhang (2008) [30] analyzed the soil aggregate distribution through the fractal method to characterize the purple soil of the Sichuan Basin and further discussed the impact of vegetation type on soil particles’ water stability, soil aggregates’ mechanics, and soil chemical stability in the Sichuan Basin. However, the volume domain fractal dimension D_v_ are obtained by laser diffraction, and the volume domain fractal dimension features of different land use types in the small watershed of purple soil have never been reported. As one of the most erodible and productive soil types in China, purple soil most distributed in southwestern China. Therefore, there is great significance to understand the response characteristics of soil fractal features.

As a new method to quantitatively describe the soil structure, the dimensionless soil fractal dimension is able to be more easily and efficiently estimated. The goal is to construct the relationship between soil PSD and the soil properties, such as soil texture, soil aggregate stability, and hydraulic conductivity. These properties will greatly affect the soil particle characteristics based on the ecological and hydrological processes in different land uses. Moreover, due to the fact that the fractal dimension is based on the self-similarity theory, which is regarded as useful way to describe the soil particle and the soil pore system [8], the fractal dimension theory is a reasonable method to evaluate the soil particle and relevant characteristics. This paper analyzes the measurement of PSD characteristics in different land uses of typical purple soil watersheds in terms of the calculation of the volume fractal dimension D_v_, including *D*_*clay*_, *D*_*silt*_, and *D*_*sand*_. The objective of this paper is to correlate the soil textures, soil organic matter, and PSD volume fractal dimension, providing the indicator for better evaluating texture, quality, and productivity of purple soil in different land uses.

## Materials and Methods

### 2.1 Study area description

**Fig 1:**
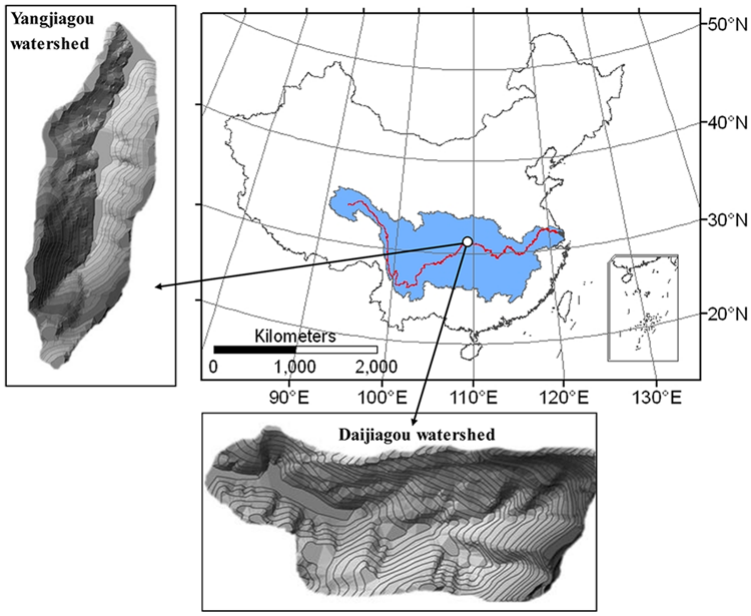
Study areas: the Daijiagou small watershed and the Yangjianggou watershed.

The study is carried out on private land of each location (Fig 1), with the permissions from the land owners. All of the land uses are either for tillage or for landscaping with economic trees, and no specific permissions were required. According to the field investigation, the field does not involve with endangered or protected species. (Fig 1, a picture shows the research areas.)

Two typical small purple soil watersheds, Yangjiagou (YJG) and Daijiagou (DJG), are chosen as the research areas. YJG is located between 108°30'18"E and 30°44'36"N, and the DJG is a small watershed located between 108°30'19"E and 30°44'21"N. The contour map in Fig 1 shows that the hillslopes of YJG and DJG, which have a south-northerly and east-westerly aspect, respectively. The areas of YJG and DJG are 60.97 hm^2^ and 66.37 hm^2^, respectively, and the altitude ranges from 422.08 m to 811.30 m for both catchments. The geomorphological compositions of the two typical purple soil dominated small watersheds mainly consist of low mountains and low hills, which are common in the Three Gorges reservoir area. Moreover, the purple soil rock types in the two study areas are mainly gray-brown purple sand mudstone of Shaximiao Formation (J_2_S). The development of the soil into a gray-brown purple soil indicates a weak erosion resistance capacity. The climates of the two research areas are mainly subtropical moist monsoon. Affected by the southeast and the southwest monsoons, and the two experimental areas have an average temperature of 17°C. The approximately annual average precipitation is 1000–1350 mm, most of which occurs during April-October. Based on the field investigation, the vegetation in the research areas mainly includes *Pinus massoniana Lamb*, *Robinia pseudoacacia L*, and *Citrus reticulate Blanco*, which represent the forest land use, and a small portion of *Zea mays L* and *Ipomoea batatas*,*and Setaria viridis*. The *Zea mays L* and *Ipomoea batatas* are the representatives of the farmland types, which are affected by human management activities, and the *Setaria viridis* represents the grassland types.

### 2.2 Soil sampling and analysis methods

Based on the field vegetation and pedologic survey of the land-use types in the YJG and DJG catchments, forestlands (*Pinus massoniana Lamb*, *Robinia pseudoacacia L*, and *Citrus reticulata Blanco*), farmlands (*Zea mays L* and *Ipomoea batatas*) and grassland (*Setaria viridis*) are chosen as the research targets. Multiple surface soil samples are collected from a depth of 20 cm in the six main vegetation areas of both catchments. The detailed soil sample collection method can be found in Zhang et al. (2008) [30–31], in which a combination of the various land-use areas in the research areas with the soil collection method is presented. The major steps of the collection method are described as follows: (1) a specific sample point in a land-use type is selected as a center point; (2) within a 10~15 m radius of this point, 4~6 surface soil samples are collected at randomly chosen locations, allowing the location is random and stochastic; and (3), these ambient point soil samples are mixed to produce an approximately 1 kg soil sample. Totally, there are 178 such soil samples are obtained from the YJG and DJG catchments.

The following analyses, which consist of three main procedures, are carried out using the soil samples.

- Dry the samples. According to experiments from Bittlli and Wang (1999, 2007) [12, 32], all the soil samples are oven-dried for 24 h at 105°C. And then, the samples are slightly crushed, and the fine remnant roots in the dry soil samples are removed. The oven-dried samples (approximately 0.50 g each) are filtered through a 2 mm sieve.
- Measure Soil PSD. The carbonates are removed from the dry soil samples using hydrochloric acid, sodium acetate, and deionized water. Peroxide (30%, w/w) is also added into the soil samples at 75°C for at least 2 h to remove the organic matter from the soil samples. And then, the acid is removed from the soil sample solution with ultra-pure water. The soil sample solution is further adjusted to a neutral pH. The samples are then immersed in [(NaPO_3_)_6_] solution for 20 h. Finally, the sample solutions are ultrasonically dispersed for 5 min and the soil PSD is calculated by *Malvern Mastersize 2000* laser diffusion (UK). The volume percentages of the soil particles are repeatedly measured by the *Mastersize 2000* based on the United States Department of Agriculture (USDA) classification of soil particle size. The soil mainly featured 7 soil particle size distributions: ultra-coarse sand (2–1 mm), coarse sand (1–0.5 mm), medium sand (0.5–0.25 mm), fine sand (0.25–0.1 mm), ultra-fine sand (0.1–0.05 mm), silt (0.05–0.002 mm), and clay (<0.002 mm).
- Calculate other soil physical and chemical properties. The soil bulk density is measured by the core cutter method (ISS, 1978), and the content of soil organic matter is determined by the potassium dichromate and sulfuric acid (ISS, 1980), which yields a value of 1.724 times that of organic carbon. The soil particle and organic matter (organic carbon) characteristics of the main land use types in the watershed are listed in Table 1.

**Table 1:** The soil texture and organic matter by land-use type.

Land-use type	Soil texture						Organic matter (g/kg)	
	Clay content		Silt content		Sand content			
	Mean (%)	CV (%)	Mean (%)	CV (%)	Mean (%)	CV (%)	Mean (%)	CV (%)
*Pinus massoniana Lamb*	3.61a	42.788	37.32c	20.485	59.07a	15.511	14.78a	43.987
*Robinia pseudoacacia L*	3.83a	25.613	42.97bc	14.518	53.20ab	13.424	13.60a	32.500
*Citrus reticulata Blanco*	3.48a	38.426	42.49bc	29.579	54.02ab	25.558	9.10b	24.477
*Zea mays L*	4.86a	26.880	54.11a	19.902	41.03c	29.390	12.07ab	36.702
*Ipomoea batatas*	3.79a	18.698	50.12ab	10.850	46.09bc	13.275	13.19ab	22.374
*Setaria viridis*	3.73a	22.854	44.07abc	18.581	52.20abc	17.192	8.73b	19.508

Footnotes: CV, coefficient of variation. Average values were analyzed by DUNCAN multiple comparisons, and different lowercase letters represent significant differences (P<0.05) between each pair.

### 2.3 Fractal dimension model for calculation

#### (1) Mass fractal dimension (D_m_) and volume fractal dimension (D_v_)

According to Yang et al. (2008) [24], the soil particle fractal dimension can be calculated in one of two ways: the soil PSD mass fractal dimension D_m_ [19] and the volume fractal dimension D_v_. Due to the assumption that the soil particles with different sizes have of the same density [22], the use of the volume fractal dimension D_v_ has been gradually adopted by other researchers. The volume-based method avoids the controversy originated from the assumption that the soil particles have self-similarity and fractal characteristics. Therefore, Wang et al. (2005) [23], further noted that D_v_ was also an intrinsic property of soil particles in analyzing the distribution of D_v_ in different land uses. Applications of the volume-based method on Yixing, Jiangsu province, China was also presented by Wang et al. (2005) [23]. Therefore, in this study, the soil particle volume fractal dimension D_v_ is chosen to evaluate the soil fractal features. The corresponding calculation model is expressed in Eq (1):

lg[V(r<Rt)VT]=(3−Dv)lg(RtRmax) (1)

Eq (1) is a double logarithmic curve function, in which *V*_*T*_ is the total volume of all soil particles, *V (r <R*_*t*_ *)* indicates the total volume of the soil particles with sizes less than the radius *R*_*t*_(mm), *R*_*max*_ is the radius of the maximal particle size, and D_v_ is the volume fractal dimension of the soil particle size distribution. In fact, the double logarithmic curves can be transformed into a linear function using lg[V(r<Rt)VT] as the y axis and $\mathrm{lg}\left(\frac{R_{t}}{R_{\max}}\right)$ as the x axis. In this case, the slope (K) of the transferred linear function can be obtained by linearly fitting to the equation k = 3-D_v_, and the volume fractal dimension D_v_ can be calculated.

#### (2) Mass domain fractal dimension (D_mi_) and volume domain fractal dimension (D_vi_)

The domain fractal dimension was developed by Bittelli et al. (1999) [12], who categorized soil particle size distribution domains primarily into clay, silt, and sand domains and discovered that the relationship between the cumulative mass of soil particles and the soil particle size does not obey strict linear relationship. Instead, only certain soil particle size domains have an obviously linear relationship between the corresponding cumulative mass and soil particle size. As a result, the fractal dimension calculation model was introduced along with three corresponding mass domain fractal dimensions (D_mi_), including D_mclay_, D_msilt_, and D_msand_ for clay, silt and sand domain, respectively. The model is expressed in Eq (2):

$${\{}_{D_{mi}=3-v}^{\frac{M\left(r<R\right)}{M_{T}}={\left(\frac{R}{R_{L.upper}}\right)}^{v}}$$ (2)

$${\{}_{D_{vi}=3-v}^{\frac{V\left(r<R\right)}{V_{T}}={\left(\frac{R}{R_{L.upper}}\right)}^{v}}$$ (3)

In Eq (2), *M (r<R)* is the mass of the soil particles a radius less than *R* (mm) and *M*_*T*_ is the total mass of particles with a radius less than the upper size limit (*R*_*L*.*upper*_) for the fractal behavior, which is determined by the measurement of specific soil samples. In fact, due to the existence of three particle size domains (clay, silt, and sand), there theoretically exists three upper sizes for soil particles of clay, silt, and sand. *v* is the constant exponent, and *D*_*mi*_ is the mass domain fractal dimension calculated individually for the clay, silt, and sand domains.

Based on Eq (2) and the theory of mass domain fractal dimension, Wang et al. (2007) [32] also reported a similar result to that from Bittelli et al. (1999) [12] with respect to the description of the PSD. Wang et al. (2007) [32] measured and analyzed the relationship between the cumulative volume of soil particles and the PSD of soil samples collected in two typical loess hilly-gullied watersheds located in Ansai county, Shaanxi province on the Loess Plateau, China. However, unlike the mass domain fractal dimension method of describing the soil PSD utilized by Bittelli et al. (1999) [12], Wang et al. (2007) [32] mainly utilized the volume domain fractal dimension (D_vi_) and Eq (3) instead of Eq (2) to describe the specific characteristics of the soil particle volume distribution.

In Eq (3), *V(r<R)* is the volume of soil particles with a radius less than *R* (mm). *V*_*T*_ is the total volume of particles with a radius less than *R*_*L*.*upper*_, and *D*_*vi*_ is the volume domain fractal dimension calculated for the clay, silt, and sand domains determined by the measurement of specific soil samples using *Malvern Mastersize 2000* laser diffusion and expressed as *D*_*vclay*_, *D*_*vsilt*_, and *D*_*vsand*_, respectively. In this paper, the three volume domain fractal dimensions are simplified as *D*_*clay*_, *D*_*silt*_ and *D*_*sand*_.

#### (3) Volume domain fractal dimension based on USDA (Dvi(U))

According to the United States Department of Agriculture (USDA) classification of soil particle size, soil particles are mainly divided into 7 partitions based on soil size. Based on the particle sizes, the clay domain is defined by particle sizes less than 0.002 mm, the silt domain is defined by particle sizes of 0.002~0.05 mm, and the sand domain particle size ranges from 0.05 mm to 2 mm. Therefore, the volume domain fractal dimension (D_vi_(U)) is the representative of *D*_*clay*_ *(U)*, *D*_*silt*_ *(U)*, and *D*_*sand*_ *(U)*, which means that the upper size limit for fractal behavior (*R*_*L*.*upper*_) described by Eq (2) is 0.002 mm, 0.05 mm, and 2 mm, respectively. Using the calculation of *D*_*silt*_ *(U)* as an example, the calculation model can be further written as Eq (4):

$$D_{silt}\left(U\right)=3-\frac{\log\left(V_{silt}+V_{clay}\right)-\log\left(V_{clay}\right)}{\log\;50-\log\;2}$$ (4)

In Eq (4), the *V*_*silt*_ and *V*_*clay*_ are the volume fraction of silt and clay with particles sizes from 0.002 mm to 0.05 mm and less than 0.002 mm, respectively. The values 50 and 2 are the upper size limit for fractal behavior *R*_*L*.*upper*_ of silt and clay, respectively, in the units of μm. Log is the natural logarithm.

To evaluate the differences between the two types of volume domain fractal dimension, as well as to assess the correlation and variability between the D_vi_ (*D*_*clay*_, *D*_*silt*_, and *D*_*sand*_) D_vi_(U) (*D*_*clay*_ *(U)*, *D*_*silt*_ *(U)*, *and D*_*sand*_ *(U)*), two statistical metrics, the correlation coefficient (R) and root mean square error (RMSE), are chosen. The calculations of R and RMSE are as shown in Eqs (5) and (6), respectively. The calculations of D_vi_ (*D*_*clay*_, *D*_*silt*_, and *D*_*sand*_) are based on the D_vi_(U) (*D*_*clay*_ *(U)*, *D*_*silt*_ *(U)*, *and D*_*sand*_ *(U)*) obtained from Eq (4).

$$R=\frac{cov\left(D_{vi},D_{vi}(U)\right)}{\sqrt{var(D_{vi})var\left(D_{vi}(U)\right)}}$$ (5)

$$RMSE=\sqrt{\frac{\sum {\left(D_{vi}-D_{vi}\left(U\right)\right)}^{2}}{N}}$$ (6)

In Eq (5), *cov(D*_*vi*,_ *D*_*vi*_ *(U))* is the covariance of *D*_*vi*_ and *D*_*vi*_ *(U)* and var(*D*_*vi*_) and var(*D*_*vi*_ *(U)*) are the variance of *D*_*vi*_ and *D*_*vi*_ *(U)*, respectively. *R* is the correlation coefficient (dimensionless). In Eq (6), *N* is the number of soil samples.

### 2.4 Statistical and other analyses

Linear regression is used to fit the volume domain fractal dimension of the soil particle distribution and the soil properties in terms of soil organic matter and soil texture. The DUNCAN significant difference analysis is also carried out to compare the six main land-use types in terms of soil properties and fractal dimensions. All statistical analyses are conducted using SPSS17.0 software.

## Results and Discussion

### 3.1 Characteristics of soil particle size distribution by land use

**Fig 2:**
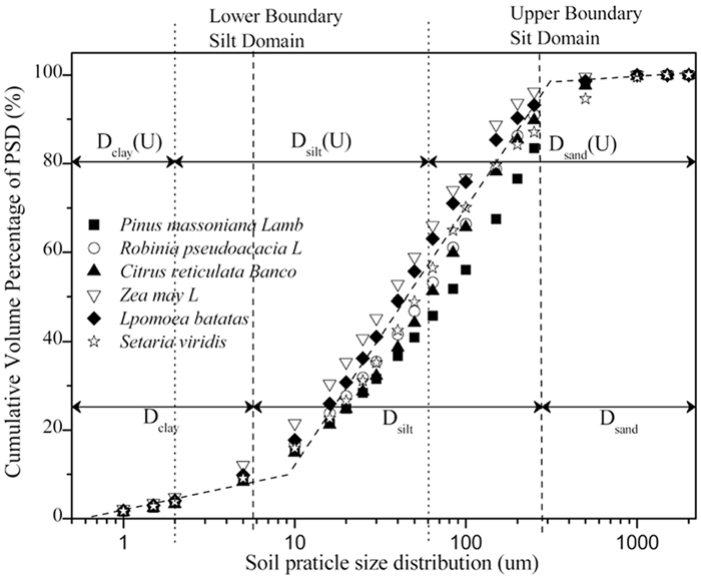
The characteristics of the soil size distribution by land-use type.

According to the analysis of the relationship between soil particle size and cumulative volume percentage distribution in six main land-use types, including *Pinus massoniana Lamb*, *Robinia pseudoacacia L*, *Citrus reticulata Blanco*, *Zea mays L*, *Ipomoea batatas*) and *Setaria viridis*, the cumulative volume percentage of soil particles and the soil particle distribution over the entire particle distribution range do not exhibit a strict linear relationship for the purple soil areas. Instead, the linear relationships between the cumulative volume percentage of soil particles and the soil particle distribution are mainly grouped into volume domains, including clay, silt, and sand domains, which is similar to the results for America, Switzerland (Bittelli et al. (1999) [12]), and the Loess Plateau of China (Wang et al. (2007) [32]). The linear relationship between soil particle size and cumulative volume percentage is shown in Fig 2. (Fig 2 shows that the linear relationship between soil particle size and cumulative volume percentage of the six land-use types, and the upper size boundaries in the measured clay domain and silt domain of purple soil were approximately 5.74μm and 286.85μm.)

However, these values are not totally in agreement with the upper size limit for fractal behavior determined by the USDA classification standard, in which the upper size limits for clay, silt, and sand domains are 0.002 mm, 0.05 mm, and 2 mm, respectively. The volume domain of the upper size limit for the fractal behavior of purple soil in typical watersheds measured by laser diffusion is also shown in Fig 2.

Moreover, the measured volume domain of the upper size boundary of purple soil is also different from the corresponding value of the for loess soil obtained by laser diffusion by Wang et al. (2007) [32]. This is mostly due to the differences of the formation and development of the solid structure of loess soil and the differences in the parent materials.

In addition, in order to obtain the best fit of the relationship between the cumulative volume percentage and the purple soil particle size in measured volume domains (*D*_*clay*_, *D*_*silt*_ and *D*_*sand*_) (i.e., an R^2^ value as calculated by Eq (5) closer to 1), the linear regression analysis method is utilized. The response characteristics of soil particle size on the cumulative volume percentages are also optimized. The fitting statistics and optimization results are shown in Table 2.

**Table 2:** Soil volume fractal dimensions and domain boundaries by land-use type.

Land-use types	D_v_		Clay domain			Silt domain			Sand domain			Median diameter		Silt domain boundary μm			
														Upper boundary		Lower	
			D_clay_			D_silt_			D_sand_			d_50_μm				boundary	
	mean	R^2^	mean	R^2^	CV (%)	mean	R^2^	CV (%)	mean	R^2^	CV (%)	mean	CV (%)	mean	CV (%)	mean	CV (%)
*Pinus massoniana Lamb*	2.478a	0.95	1.910c	0.98	0.572	2.487ab	0.98	3.722	2.960ab	0.91	0.941	77.44a	35.331	286.966a	0.003	5.749a	0.003
*Robinia pseudoacacia L*	2.493a	0.92	1.921c	0.99	0.461	2.485ab	0.99	2.625	2.985a	0.90	0.460	58.06a	27.213	286.960a	0.001	5.743a	0.002
*Citrus reticulata Blanco*	2.470a	0.93	1.937bc	0.97	2.560	2.429b	0.98	4.857	2.950bc	0.92	1.354	61.91ab	41.095	286.958a	0.001	5.749a	0.003
*Zea mays L*	2.533a	0.90	1.933bc	0.98	0.348	2.531a	0.98	3.193	2.982ab	0.89	0.322	39.89b	39.142	286.852b	0.008	5.748a	0.017
*Ipomoea batatas*	2.501a	0.91	1.960ab	0.96	0.292	2.483ab	0.97	2.796	2.975ab	0.90	0.278	42.34b	24.370	286.877b	0.088	5.748a	0.020
*Setaria viridis*	2.489a	0.92	1.969a	0.96	0.981	2.462ab	0.98	1.151	2.925c	0.92	1.580	60.08ab	39.382	286.960a	0.001	5.749a	0.002

Footnotes: CV, coefficient of variation.

As shown in Fig 2, differs from the USDA classification standard, the upper size boundaries in the measured clay and silt domain of purple soil for the six land-use types of the two typical small watersheds are approximately 5.74μm and 286.85μm, respectively. Moreover, Table 2 shows that, among the different land-use types, the volume domain upper size of clay ranges from 5.743μm to 5.749μm and the mean volume domain upper sizes for different land uses are not significantly different from each other (P<0.05). The upper size boundary in the measured silt domain of purple soil is 286.852~286.966 μm. Additionally, based on the determination of the volume fractal dimension calculated by Eq (1), the volume fractal dimension (D_v_) and volume domain fractal dimension (D_vi_) are calculated and the results are shown in Table 2. The results show that among the six various land-use types, the mean of D_v_ fluctuates between 2.470 and 2.533 without a significant difference (P<0.05) as it is compared with D_vi_.

More specific information is also provided in Table 2. First, the calculated mean *D*_*clay*_ for all land uses using the laser diffusion instrument ranges from 1.910 to 1.969. The corresponding determination coefficient reaches 0.97, which indicates that the fitting of the relationship between the cumulative volume percentage of soil particles and soil particle distribution is reasonably good. The average *D*_*clay*_ values for different land use types have the following relationships: *Setaria viridis*>*Ipomoea batatas>Citrus reticulata Blanco>Zea mays L>Robinia pseudoacacia L*>*Pinus massoniana Lamb*. Wang et al. (2005) and Konert et al. (1997) reported that as it was compared with the volume contents of clay and silt measured by the pipette method [33], the values obtained using laser diffusion (specifically, by the application of *Malvern Mastersize 2000* laser diffusion) were lower and higher, respectively. And the *D*_*clay*_ for loess soil was even negative (Wang et al. (2005) [23]). However, in this paper, the value of *D*_*clay*_ for different land uses in two typical purple soil from the small watersheds is higher than what Wang et al. (2005) and Konert et al. (1997) reported. The average value of *D*_*clay*_ in our experiment reaches nearly 1.94, which is much greater than the value for loess soil. The *D*_*clay*_ differences between purple and loess soil are most likely due to the variability of the parent material, which is an important soil-forming factor affecting the soil physical and chemical properties. In fact, due to the higher weathering rate of the parent material of purple soil, the proportion of fine particles in the soil PSD becomes very high. The soil-forming periodicity of purple soil from parent material weathering to the mature soil formation is shorter than other soil type. Therefore, the average value of *D*_*clay*_ in purple soil is positive and even higher than loess soil.

According to the analysis and calculation of *D*_*silt*_ and *D*_*sand*_ by land use, the *D*_*silt*_ and *D*_*sand*_ of *Zea mays L* and *Ipomoea batatas*, *Robinia pseudoacacia L* and *Pinus massoniana Lamb* are all higher than the values of *D*_*silt*_ and *D*_*sand*_ in *Setaria viridis* and *Citrus reticulate Blanco* land uses located in the two typical purple soil watersheds. All of these values are similar to the results of the volume fractal dimension analysis on soil PSD under different land uses in the Loess Plateau by Wang et al. (2007) [32]. The higher *D*_*silt*_ and *D*_*sand*_ in *Zea mays L* and *Ipomoea batatas* land use indicate that fine soil particles are more common in the silt domain and sand domain. The higher values of *D*_*silt*_ and *D*_*sand*_ in *Zea mays L* and *Ipomoea batatas* land are most likely due to the human activities, such as tillage, cultivation, and fertilization. These activities are able to crush coarse soil particles into fine particles while improve the nutrient utilization of vegetation planted in these land uses.

The higher values of *D*_*silt*_ and *D*_*sand*_ for the soil samples collected in the *Robinia pseudoacacia L* and *Pinus massoniana Lamb* land uses indicate a higher proportion of fine particles in the silt and sand domains in typical forestland. The deep-root characteristics, which also play a pivotal role in improving soil structure, have greatly influenced the soil porosity and the quantity of soil microorganism. In addition to the effect of deep-root systems on the soil solid properties, the soil particle and nutrients, especially on the surface layer of the soil profile, are affected by the litter layer covering the forest soil. Therefore, distinct from reasons for the *Zea mays L* and *Ipomoea batatas* land, the plant physiological characteristics are most likely the main causes of the higher values of *D*_*silt*_ and *D*_*sand*_ for the forestland (*Robinia pseudoacacia L* and *Pinus massoniana Lamb*).

Moreover, regarding the lower *D*_*silt*_ and *D*_*sand*_ values calculated from the soil sampled with the *Setaria viridis* and *Citrus reticulate Blanco* land uses, *Setaria viridis* has a poor capability to conserve the soil structure due to its shallow-root physiological characteristics. In addition, large amounts of *Setaria viridis* in both of the two selected typical purple soil small watersheds are distributed on steep hillslopes and poor nutrient areas, which are able to influence the formation and the accumulation of the fine soil particles. There covers a thin layer of *Citrus reticulate Blanco*, which is an important plant introduced in the Grain-for-Green Project for both ecological and economic benefits in many purple soil watershed areas in southwestern China. The *Citrus reticulate Blanco* is able to protect many fine soil particles from being transported away while leaving coarser particles by the exceeded the runoff caused by the intensified precipitation over the threshold.

According to a comparison of the volume domain fractal dimension in different land uses, the value of D_vi_ followes the sequence of *D*_*clay*_ *<D*_*silt*_ *<D*_*sand*_. This relationship is similar to the results from Bittelli et al. (1999). We also find that the average value of *D*_*v*_ is close to the average value of *D*_*silt*,_ and the absolute value of the difference between *D*_*v*_ and *D*_*silt*_ is 0.008~0.041. The higher determination coefficient (0.89≤R^2^≤0.99) shown in Table 2 indicates that the volume domain fractal dimension model is able to efficiently describe the volume distribution of the soil particle size. The determination coefficient of *D*_*silt*_ is higher than the determination coefficient of *D*_*clay*_, and the *D*_*sand*_ is similar to the results from Bittelli et al. (1999) The reason to explain this fact was also given by Bittelli et al. (1999), in which the authors noted that based on the measurement of 19 soil samples from American and Switzerland, the classification and data acquisition for the silt and sand domains were most likely affected by the limitation of the experiments in terms of testing the soil PSD using the *Malvern Mastersize 2000*. More specifically, the error in the sand domain classification was mainly due to the processes of soil particle sieving; however, the limitations and accuracy of the silt domain classification were strongly affected by the application of laser diffusion technology, all of which may cause the difference between *D*_*clay*_, *D*_*silt*_, and *D*_*sand*_.

### 3.2 Comparison of the responses of *D*_*vi*_ and *D*_*vi*_ *(U)*by land use

**Fig 3:**
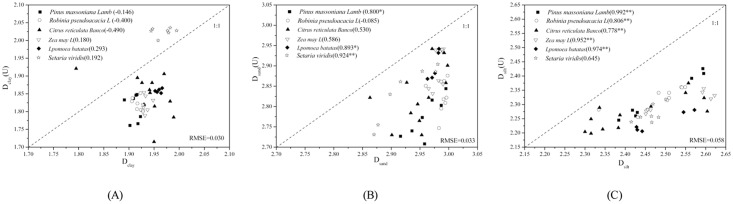
The comparison of the volume domain fractal dimension based on the measured D_vi_ and volume domain fractal dimension using USDA (D_vi_(U)) land-use type classification. Footnote: the number in brackets represents the correlation coefficient; * indicates significant correlation (P<0.05), ** indicates significant correlation (P<0.01).

Based on Eqs (3)–(6), the relationship between *D*_*vi*_ and *D*_*vi*_ *(U)* is shown by different classification standards in Fig 3. According to the *D*_*vi*_ and *D*_*vi*_ *(U)* in the clay domain (Fig 3), the *D*_*clay*_ values are larger than the corresponding *D*_*clay*_ *(U)* values (except for the *D*_*clay*_ of *Setaria viridis*, which is smaller than the corresponding *D*_*clay*_ *(U)*). The RMSE shown in Fig 3(a) is 0.030. Similarity, in the silt domain, the *D*_*silt*_ values of all six land-use types are consistently higher than the corresponding *D*_*silt*_ *(U)* with a regularity that was also found for *D*_*sand*_ and *D*_*sand*_ *(U)* for all six land-use types. Generally, the higher value of *D*_*vi*_ with regard to *D*_*vi*_ *(U)* are affected by both of the classifications types and the volume domains standards. Moreover, according to the results from Bittelli et al. (1999), in which the RMSE of *D*_*silt*_ and *D*_*silt*_ *(U)* were 0.090 for soil from America and Switzerland. This value is larger than that of *D*_*silt*_ and *D*_*silt*_ *(U)* for soil from two typical purple soil small watersheds (0.058) in our experiments. The difference in the RMSE of *D*_*silt*_ and *D*_*silt*_ *(U)* can be explained with the following two reasons. On one hand, the calculation of *D*_*vi*_ and *D*_*vi*_ *(U)* by Bittelli et al. (1999) is mainly based on Eq (2), which essentially represents the mass domain fractal dimension (D_mi_), rather than the volume domain fractal dimension (D_vi_). In our experiments, Eq (3) is used to describe the volume fractal behavior of purple soil. On the other hand, the soil samples collected by Bittelli et al. (1999) were mainly derived from 7 parent materials, with the heterogeneity of soil properties. However, the parent material for the purple soil develops from gray-brown purple sand mudstone of the Shaximiao Formation (J_2_S). Therefore, due to the relative homogeneity of the purple soil parent materials, the RMSE of *D*_*silt*_ and *D*_*silt*_ *(U)* is higher than what Bittelli et al. (1999) reported. (Fig 3 shows the correlation analysis for Dvi and Dvi(U) for different land uses in typical purple soil small watershed, revealing that the correlation between Dclay and Dclay(U) of all six land-use types was not significant.)

Fig 3 also shows the correlation analysis for *D*_*vi*_ and *D*_*vi*_ *(U)*for different land uses in typical purple soil in the selected small watersheds. Generally, the correlations between *D*_*clay*_ and *D*_*clay*_ *(U)* of all six land-use types are not significant. However, in the sand domain, the correlation between *D*_*sand*_ and *D*_*sand*_ *(U)* for the *Pinus massoniana Lamb* (R = 0.800), *Ipomoea batatas* (R = 0.893), and *Setaria viridis* (R = 0.924) land uses are significant. In the silt domain, except for the correlation between *D*_*silt*_ and *D*_*silt*_ *(U)* of *Setaria viridis* land use, the correlations between *D*_*silt*_ and *D*_*silt*_ *(U)* of the other five land-use types are significant and positive. The correlation coefficient of *Pinus massoniana Lamb* land use is the highest (0.992) among all types, and the lowest correlation coefficient (0.778) appears in case of the *Citrus reticulate Blanco* land use.

Based on the significant correlation between *D*_*vi*_ and *D*_*vi*_ *(U)* for different land uses in a typical purple soil in the selected small watershed, the reasons that the correlation between *D*_*silt*_ and *D*_*silt*_ *(U)* is more significant than that between *D*_*sand*_ and *D*_*sand*_ *(U)* and between *D*_*clay*_ and *D*_*clay*_ *(U)* is listed below: Both of the ranges of *D*_*silt*_ and *D*_*silt*_ *(U)* have wider soil PSD in the silt domain than the clay and sand domains. A wider soil PSD means that more soil particle size samples are included and contained in the wider distribution range, which is able to more efficiently and accurately represent all of the characteristics of the volume soil particle distribution relative to the clay and sand domains. Therefore, the correlation between *D*_*silt*_ and *D*_*silt*_ *(U)* is more significant than that with clay and sand.

### 3.3 Response of volume domain fractal dimensions to the soil properties

**Fig 4:**
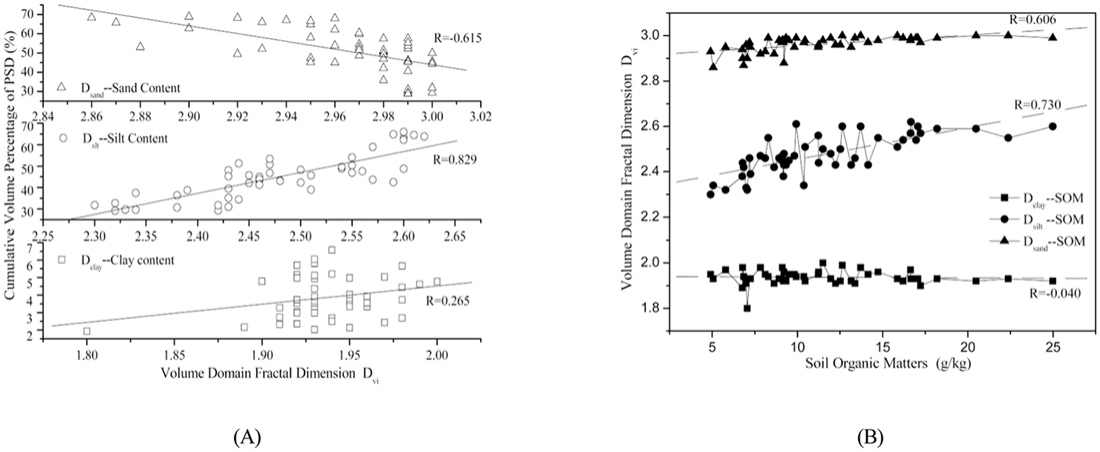
The correlation between volume domain fractal dimension based on measured D_vi_ and soil texture (A) and linear regression analysis for D_vi_ and soil organic matter (B) on a watershed scale.

The response of volume domain fractal dimensions to soil properties, i.e., soil texture and soil organic matter, is shown in Table 3 and Fig 4. The significance of the correlations between *D*_*silt*_, *D*_*silt*_ *(U)*, *D*_*sand*_, *D*_*sand*_ *(U)*, and *D*_*clay*_ and soil texture differs from each other, except for the insignificant correlation between *D*_*clay*_ *(U)* and soil clay, silt, and sand contents. The correlations between the soil organic matter and the volume domain fractal dimensions are significant to different extents, which includes *D*_*clay*_ *(U)*, *D*_*silt*_, *D*_*silt*_ *(U)*, and *D*_*sand*_ with the corresponding correlation coefficients of -0.420, 0.730, 0.621, and 0.606, respectively. (Fig 4, in the both typical purple soil small watersheds, except for the insignificant correlation between Dclay(U)and soil clay, silt, and sand contents, the significance of the correlations between Dsilt, Dsilt(U), Dsand, Dsand(U), and Dclay and soil texture differed. On the other hand, the correlativity of soil organic matter and volume domain fractal dimensions were significant to different extents.)

**Table 3:** The correlation analysis between Dvi (Dvi(U)) and soil texture and organic matter on a watershed scale.

Classification scale	Soil properties	D_v_	Clay domains		Silt domains		Sand domains	
			D_clay_	D_clay_(U)	D_silt_	D_silt_(U)	D_sand_	D_sand_(U)
Watershed scale	Clay content	0.816**	—	—	0.913**	0.723**	0.604**	0.582**
	Silt content	0.735**	0.420**	—	0.829**	0.441**	0.606**	0.689**
	Sand content	-0.748**	-0.406**	—	-0.846**	-0.480**	-0.615**	-0.593**
	Organic matter	0.564**	—	-0.420**	0.730**	0.621**	0.606**	—

Footnotes:

**, significant correlation (P<0.01);

—, no significant correlate

**Fig 5:**
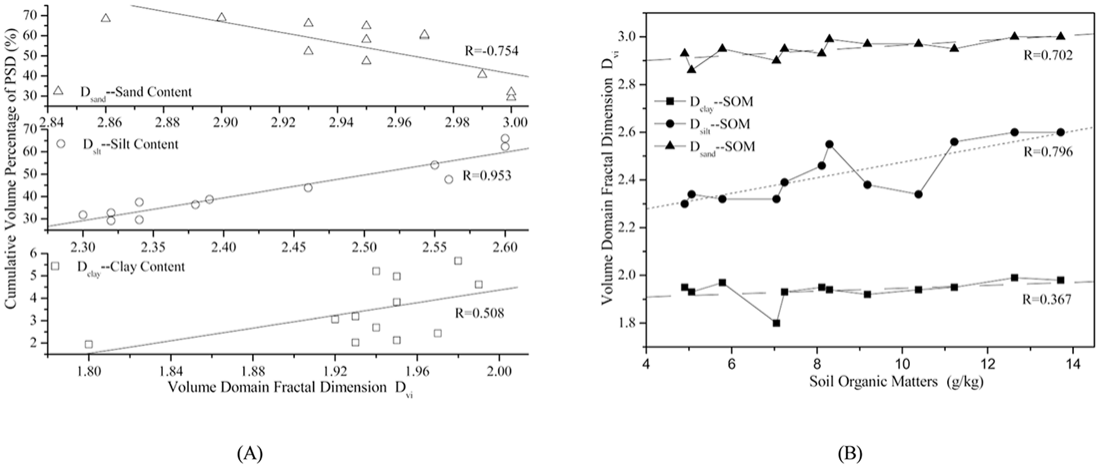
The correlation between volume domain fractal dimension based on the measured D_vi_ and soil texture (A) and linear regression analysis for D_vi_ and soil organic matter (B) for the *Citrus reticulata Blanco* land-use type.

The correlation between the soil properties in terms of soil texture as well as the organic matter and volume domain fractal dimension in terms of *D*_*vi*_ and *D*_*vi*_ *(U)* is analyzed. The results are shown in Table 4. Specifically, the correlation analysis is conducted between the soil texture, including clay, silt and sand content for different land uses, and their corresponding volume domain fractal dimensions, including *D*_*clay*_, *D*_*silt*_, and *D*_*sand*_, in the typical purple soil for the selected small watersheds. In addition, the correlation between the soil organic matter and volume (*D*_*vi*_ and *D*_*vi*_ *(U)*) is also analyzed (Table 4). Moreover, using the soil properties (soil texture and organic matter) for the *Citrus reticulate Blanco* land use as an example, these correlations between *D*_*vi*_ (*D*_*clay*_, *D*_*silt*_ and *D*_*sand*_) are also obtained. A linear regression analysis is carried out to fit the characteristics of volume domain fractal dimension and corresponding soil properties, such as soil texture and organic matter. The fitting results are shown in Fig 5. (Fig 5, analysis of the correlation between Dvi(Dclay, Dsilt and Dsand) and soil properties (soil texture and organic matter) in Citrus reticulate Blanco land use, these correlations were also described using linear regression analysis to fit the characteristics of volume domain fractal dimension and corresponding soil properties (soil texture and organic matter).)

**Table 4:** The correlation analysis between D_vi_ and D_vi_(U) and soil texture and organic matter for six land-use types.

Land-use type	Soil properties	D_vi_	Clay domains		Silt domains		Sand domains	
			D_clay_	D_clay_(U)	D_silt_	D_silt_(U)	D_sand_	D_sand_(U)
*Pinus massoniana Lamb*	Clay content	0.980**	—	-0.907**	0.989**	0.971**	0.874*	0.832*
	Silt content	0.953**	—	-0.805*	0.949**	0.922**	0.874*	—
	Sand content	-0.927**	—	0.927**	-0.956**	-0.914**	-0.912**	-0.861*
	Organic matter	0.935**	—	—	0.939**	0.897**	0.904**	0.778*
*Robinia pseudoacacia L*	Clay content	0.871**	—	-0.783**	0.873**	0.810**	—	—
	Silt content	0.895**	0.705*	-0.700*	0.921**	0.783**	0.720*	—
	Sand content	-0.901**	-0.693*	0.719*	-0.924**	0.795**	-0.712*	—
	Organic matter	0.862**	—	-0.715*	0.804**	0.761*	0.648*	—
*Citrus reticulata Blanco*	Clay content	0.910**	—	—	0.964**	0.863**	0.705*	0.614*
	Silt content	0.848**	—	—	0.953**	0.690*	0.753**	—
	Sand content	-0.860**	—	—	-0.961**	-0.712**	-0.754**	-0.581*
	Organic matter	0.702*	—	—	0.976**	0.645*	0.702*	—
*Zea mays L*	Clay content	0.986**	—	—	0.978**	0.899**	0.856**	—
	Silt content	0.972**	—	—	0.987**	0.949**	0.907**	—
	Sand content	-0.983**	—	—	-0.988**	-0.945**	-0.904**	—
	Organic matter	0.783*	—	—	0.781*	0.885**	0.766*	—
*Ipomoea batatas*	Clay content	—	—	—	—	—	—	—
	Silt content	—	—	0.906*	—	—	—	—
	Sand content	—	—	-0.894*	—	—	—	—
	Organic matter	—	—	—	—	—	—	—
*Setaria viridis*	Clay content	0.766*	—	—	0.928	—	0.810*	—
	Silt content	—	—	—	0.757*	—	0.826*	0.775*
	Sand content	—	—	—	-0.779*	—	-0.831*	-0.773*
	Organic matter	0.799*	—	—	0.890**	—	—	—

Footnotes:

*, significant correlation (P<0.05);

**, significant correlation (P<0.01);

—, no significant correlation.

Table 4 shows that the *D*_*vi*_ and soil properties are significantly correlated, except for the cases of the *Ipomoea batatas* and *Setaria viridis* land uses. The correlations are especially significant for the *Robinia pseudoacacia L* and *Pinus massoniana Lamb* land uses. Moreover, Table 4 also shows that the correlation between *D*_*silt*_ and soil properties is significant for five of the land uses (*Pinus massoniana Lamb*, *Robinia pseudoacacia L*, *Citrus reticulata Blanco*, *Zea mays L*, *and Setaria viridis*), which is similar to the correlativity between *D*_*sand*_ and soil properties. However, the correlativity between *D*_*clay*_ and soil texture and organic matter is not significant for all land uses. Additionally, in the cases that the calculation of the *D*_*vi*_ *(U)* is based on the USDA classification and standards. Table 4 also indicates that the correlation between *D*_*clay*_ *(U)* and soil texture and organic matter is not significant, neither does the sand domain. However, in the silt domain, the correlations are significant for four land uses: *Pinus massoniana Lamb*, *Robinia pseudoacacia L*, *Citrus reticulata Blanco*, and *Zea mays L*. Table 4 also indicates that the correlation between sand content and all the volume domain fractal dimensions, including *D*_*vi*_ and *D*_*vi*_ *(U)*, is significant and negative correlated, which is similar to the results from Wu et al.(1993), Yang et al.(2008), and Wang et al(2005). [10,23–24].

Additionally, as it is shown in Table 3, the *D*_*silt*_, *D*_*silt*_ *(U)*, *D*_*sand*_, and *D*_*sand*_ *(U)* have stronger correlativity with soil texture and show more obvious responses to the soil property, comparing with that in the clay domain (*D*_*clay*_). The soil texture is ranked as the following: *D*_*silt*_>*D*_*silt*_ *(U)>D*_*sand*_ *(U)*>*D*_*sand*_. The responses of *D*_*clay*_ and *D*_*clay*_ to the soil organic matter in all the land-use types are weak due to the poor correlation. However, the *D*_*silt*_, *D*_*silt*_ *(U)*, *D*_*sand*_, and *D*_*sand*_ *(U)* are positively correlated to varying extents of the soil organic matters. The intensity of the response of the volume domain fractal dimension to the soil organic matter has the following order: *D*_*silt*_>*D*_*silt*_ *(U)*>*D*_*sand*_ *>D*_*sand*_ *(U)*.

**Fig 6:**
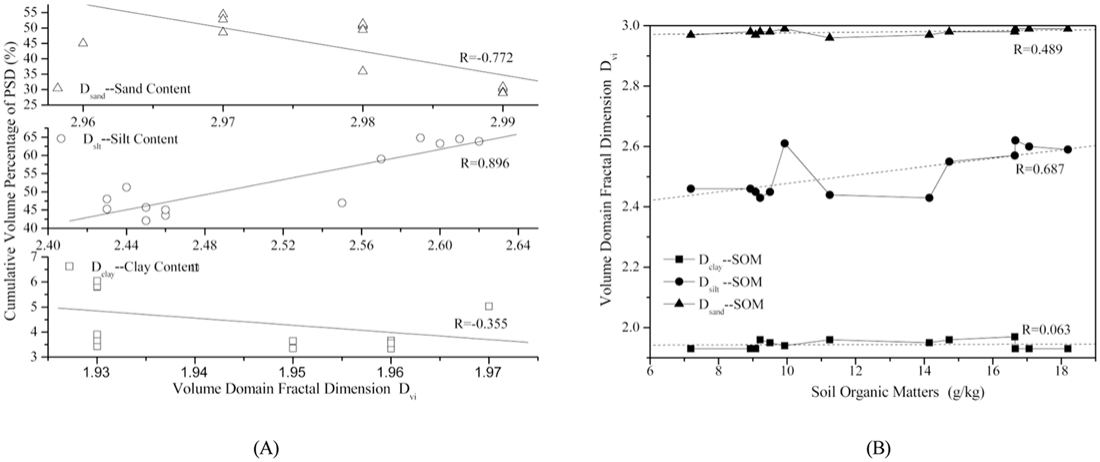
The correlation between volume domain fractal dimension based on the measured *D*_*vi*_ and soil texture (A) and linear regression analysis for *D*_*vi*_ and soil organic matter (B) for the agriculture land-use type.

In addition, regarding the response of volume domain fractal dimension to the soil properties in different land uses of the two typical purple soil small catchments, the six land uses are classified into two groups based on their functionalities: forestland, which is the representative of *Robinia pseudoacacia L*, *Pinus massoniana*, and *Citrus reticulata Blanco*, and farmland, which is the representative of *Zea mays L* and *Ipomoea batatas*. Based on classification of the land uses, Table 5 shows the response of the volume domain fractal dimension of forestland and farmland to the soil properties (soil texture as well as the organic matter) in terms of the correlation coefficients for different volume domain fractal dimensions and the soil texture and soil organic matter. Moreover, for the example of agricultural land, the relationship between *D*_*vi*_ and soil texture and organic matter are presented using a linear fitting analysis, as it is shown in Fig 6. (Fig 6, the relationship between Dvi and soil texture and organic matter were subjected to linear fitting analysis on the agricultural land.)

**Table 5:** The correlation analysis between *D*_*vi*_ and *D*_*vi*_ *(U)* and soil texture and organic matter in the forestland and the agricultural land types.

Land-use type	Soil properties	D_v_	Clay domains		Silt domains		Sand domains	
			D_clay_	D_clay_(U)	D_silt_	D_silt_(U)	D_sand_	D_sand_(U)
Forestland(P/R/C)	Clay content	0.924**	0.373*	-0.505**	0.921**	0.840**	0.674**	0.526**
	Silt content	0.829**	0.551**	-0.437**	0.844**	0.581**	0.681**	0.587**
	Sand content	-0.844**	-0.534**	0.461**	-0.864**	-0.625**	-0.695**	-0.604**
	Organic matter	0.714**	—	-0.498**	0.762**	0.708**	0.642**	—
Agricultural land(Z/L)	Clay content	0.891**	—	-0.638*	0.917**	0.828**	0.803**	—
	Silt content	0.887**	—	—	0.896**	0.703**	0.764**	—
	Sand content	-0.896**	—	—	-0.903**	-0.721**	-0.772**	—
	Organic matter	0.678*	—	—	0.687**	—	—	—

Footnotes:

**, significant correlation (P<0.01);

—, no significant correlation.

P, R, C, Z, and L represent *Pinus massoniana Lamb*, *Robinia pseudoacacia L*, *Citrus reticulata Blanco*, *Zea mays L*, and *Ipomoea batatas*, respectively.

From Table 4, the intensity of the response of the volume domain fractal dimension to the soil texture for the forestland has the following order: *D*_*silt*_>*D*_*silt*_ *(U)*>*D*_*sand*_ *>D*_*sand*_ *(U)*>*D*_*clay*_ *>D*_*clay*_ *(U)*. The correlation coefficients between the volume domain fractal dimension and soil organic matter for *D*_*silt*_ *(U)*, *D*_*sand*_, and *D*_*clay*_ *(U)*, are 0.708, 0.642, and -0.498, respectively. The largest correlation coefficient appears in the soil type of silt. In the agricultural land, including *Zea mays L* and *Ipomoea batatas*, only *D*_*silt*_, *D*_*silt*_ *(U)*,and *D*_*sand*_ have significant correlations with the soil texture. However, only in forestland, *D*_*silt*_ has a significant correlation (R = 0.687) between volume domain fractal dimension and soil organic matter.

**Fig 7:**
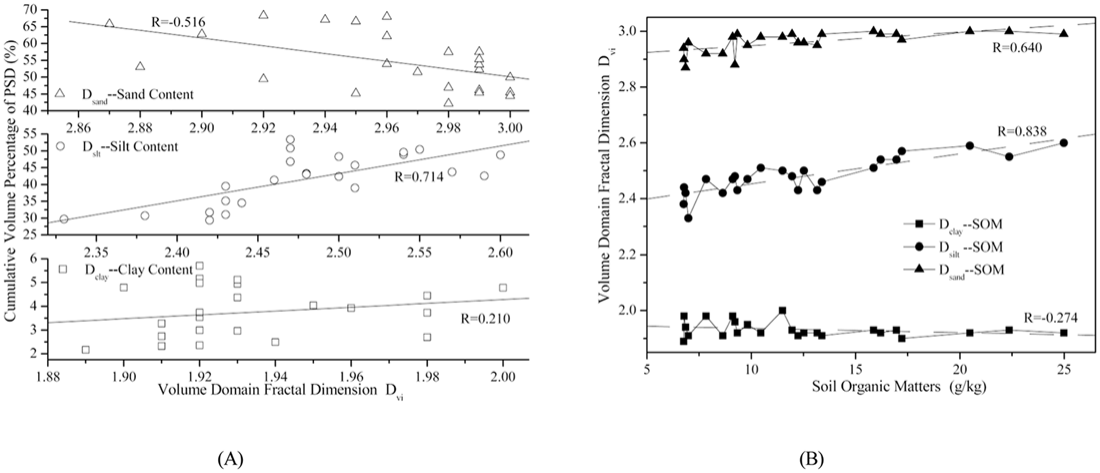
The correlation between the volume domain fractal dimension based on the measured *D*_*vi*_ and soil texture (A) and linear regression analysis for *D*_*vi*_ and soil organic matter (B) for group I land use.

The influence of human activities, such as tillage, fertilization, and other soil management practices, on the response of the volume domain fractal dimensions to the soil properties is analyzed. To better illustrate the difference, we group the land-use types based on their intensity of exposure to human activities. *Pinus massoniana Lamb*, *Robinia pseudoacacia L*and *Setaria viridis* are categorized into the group I landuse, which is not affected by intensive human activities. The group II land use consists of *Zea mays L*, *Ipomoea batatas*, and *Citrus reticulate Blanco* land uses, all of which are intensively affected by human. The correlation test and a linear regression analysis are carried out. The results for both of the two groups are shown in Table 6 and Fig 7. (Fig 7, group I has a similar correlation between volume domain fractal dimensions, and the correlativity of Dsilt between soil organic matter was most significant in the corresponding groups.)

**Table 6:** The correlation analysis between *D*_*vi*_ and *D*_*vi*_ *(U)* and soil texture and organic matter for group I and II land uses.

Land-use type	Soil properties	D_v_	Clay domains		Silt domains		Sand domains	
			D_clay_	D_clay_(U)	D_silt_	D_silt_(U)	D_sand_	D_sand_(U)
Group I(P/R/S)	Clay content	0.908**	—	—	0.907**	0.777**	0.531**	0.519**
	Silt content	0.800**	0.413*	—	0.714**	0.468*	0.494*	0.683**
	Sand content	-0.814**	—	—	-0.807**	-0.542**	-0.561**	-0.698**
	Organic matter	0.708**	—	-0.617**	0.838**	0.806**	0.640*	—
Group II(C/Z/L)	Clay content	0.921**	—	-0.438*	0.943**	0.821**	0.690**	0.636**
	Silt content	0.889**	0.399*	-0.472*	0.944**	0.666**	0.757**	0.647**
	Sand content	-0.898**	—	0.472*	-0.949**	-0.686**	-0.755**	-0.650**
	Organic matter	0.725**	—	—	0.755**	0.505*	0.632**	0.573**

Footnotes:

**, significant correlation (P<0.01);

—, no significant correlation.

P, R, C, Z, L, and S represent *Pinus massoniana Lamb*, *Robinia pseudoacacia L*, *Citrus reticulata Blanco*, *Zea mays L*, *Ipomoea batatas*, and *Setaria viridis*, respectively.

As it is shown in Table 5, the correlation between *D*_*clay*_ and soil texture in group II is not significant. The other volume domain fractal dimensions, including *D*_*silt*_, *D*_*silt*_ *(U)*, *D*_*sand*_, *D*_*sand*_ *(U)*, and *D*_*clay*_ *(U)*, have different extents of significant correlations with soil texture. The intensity of the response of these volume domain fractal dimensions to the soil texture has the following order: *D*_*silt*_>*D*_*silt*_ *(U)*>*D*_*sand*_ *>D*_*sand*_ *(U)>D*_*clay*_ *(U)*. In contrast, group I has a similar correlation between volume domain fractal dimensions, including *D*_*silt*_, *D*_*silt*_ *(U)*, *D*_*sand*_, *and D*_*sand*_ *(U)*, and soil texture. Moreover, in both group I and group II, the correlativity of *D*_*silt*_ between soil organic matter is the most significant. The correlation coefficients reach 0.838 and 0.755 for group I and II, respectively.

This method to group the land use types yields a correlation between *D*_*silt*_ and soil properties in terms of soil texture and organic matter, both of which are the key representatives of the indicators of soil quality [34]. This correlation is the most significant under the purple soil condition. It indicates that the volume domain fractal dimensions, especially *D*_*silt*_, are able to be used as potential indicators of the soil texture and soil productivity. Moreover, given the fact that there is a significant positive correlativity between *D*_*silt*_ and the silt and clay content, as well as the fact that there exists a significant negative correlativity between *D*_*silt*_ and sand content, it is able to conclude that, the *D*_*silt*_, as a volume domain fractal dimension, reflects not only the degree of fragmentation of soil particles but also the characteristics of the soil organic matter coupling with the soil particles in purple soil. Therefore, *D*_*silt*_ is more suitable than any other mass or volume domain fractal dimensions for describing and evaluating the characteristics of the relationship between soil texture, organic matter, and soil particles.

## Conclusion

The conclusions of the response characteristics of soil fractal features by land use in a typical purple soil watershed are summarized below:

The volume domain upper size of the clay domain ranges from 5.743μm to 5.749μm for all land-use types, and the boundary of the upper size of the silt domain for purple soil is 286.852~286.966 μm. In all land-use types under the purple soil condition, the volume domain fractal dimensions have the following order: *D*_*clay*_ *<D*_*silt*_ *<D*_*sand*_. Regarding the land uses, the values of *D*_*silt*_ and *D*_*sand*_ in *Citrus reticulate Blanco* and *Setaria viridis batatas* are smaller than the corresponding values in *Pinus massoniana Lamb*, *Robinia pseudoacacia L*, and *Ipomoea*. In addition, for all land-use types, all of the parameters in D_vi_ *(D*_*clay*_, *D*_*silt*_, and *D*_*sand*_) are higher than the corresponding parameters in D_vi_(U) *(D*_*clay*_ *(U)*, *D*_*silt*_ *(U)*,and *D*_*sand*_ *(U)*). Moreover, regarding the response of the volume domain fractal dimension to the soil properties, the strengths of the correlation to the soil texture and soil organic matter have the following ranks: *D*_*silt*_>*D*_*silt*_ *(U)>D*_*sand*_ *(U)*>*D*_*sand*_ and *D*_*silt*_>*D*_*silt*_ *(U)*>*D*_*sand*_ *>D*_*sand*_ *(U)*, respectively. Finally, due to the fact that *D*_*silt*_ has the most significant correlativity to the soil texture and organic matter among the various land uses of typical purple soil watersheds, it can be regarded as a potential indictor for evaluating the proportion of fine particles in PSD, as well as a key measurement for soil quality and productivity studies.
